# Evaluating the Impact of a Targeted Approach Designed to Build Executive Function Skills: A Randomized Trial of Brain Games

**DOI:** 10.3389/fpsyg.2021.655246

**Published:** 2021-10-01

**Authors:** Sophie P. Barnes, Rebecca Bailey, Stephanie M. Jones

**Affiliations:** Harvard Graduate School of Education, Cambridge, MA, United States

**Keywords:** executive functions, self-regualtion, classroom and school based research, classroom intervention, school-aged children

## Abstract

This paper reports results from an impact study of Brain Games (BGs), a classroom-based intervention designed to build preschool and school-aged children’s executive functions (EFs) and related self-regulation skills. The study employed a classroom-randomized, experimental design with 626 students in 36 pre-K through fourth grade classrooms in charter schools in a mid-sized urban district. In one set of models with child covariates, children in intervention classrooms showed marginal positive impacts on regulation-related behaviors, attention control and impulsivity, and negative effects on global EF and marginal increases in discipline problems. A second set of models with a smaller sample and both child and classroom covariates included indicate positive impacts of BGs on global EFs, prosocial behavior, and attention control and impulsivity. There were no significant impacts on the teacher–student relationship as reported by the teacher or on direct assessments of inhibitory control, short term and working memory, or another measure of global EF in either set of models. These promising findings offer a signal that implementation of targeted, easy to implement intervention approaches in classroom contexts can influence children’s regulation-related and prosocial outcomes, but this signal should be investigated further with larger and more tightly controlled designs.

## Introduction

Over the past several decades, a robust body of literature has emerged documenting the foundational role of social and emotional skills for learning, behavior, and health (Durlak et al., 2011; Moffitt et al., 2011; Greenberg et al., 2017; Jones and Doolittle, 2017). Long-term correlational studies document that social and emotional skills in childhood such as social competence and self-control are associated with important life outcomes 20–30years later including labor market success, higher education, physical and mental health, low substance use, personal finances, and low criminal offending (Moffitt et al., 2011; Jones D. et al., 2015, 2019b). Gaining increasing attention and investment in the research (e.g., this special issue; Gates and Chan-Zuckerberg jointly funded the EF+Math 5-year grant program designed to fund multi-disciplinary teams who will integrate EFs into high-quality math), practice (e.g., focus on EFs and self-control in the KIPP model and the Head Start Early Learning Outcomes Framework), and policy communities (e.g., see Osher et al., 2016; Jones et al., 2019a), efforts to educate the “whole child” are increasingly central to children’s school experiences. School-based programs, curricula, and interventions are now widely adopted in school contexts, though an array of challenges related to feasibility of implementation and the challenges of integration in the instructional work of classrooms remain (Jones and Bouffard, 2012; Jones et al., 2018).

Children’s development across social, emotional, behavioral, and cognitive domains is posited to build on a set of core executive functions and self-regulation skills (EFs and SR; Diamond and Lee, 2011; Jones et al., 2016; Bailey and Jones, 2019; Diamond and Ling, 2019). Beginning in early childhood, these foundational skills support the emergence and growth of more complex skills and competencies. For example, basic EFs and SR skills enable children to learn how to inhibit impulses and take turns, which undergird basic prosocial behavior and cooperation, and later more complex social problem solving. Myriad examples demonstrate the foundational and significant role of EFs and SR in children’s development (e.g., Center on the Developing Child at Harvard University, 2011; Bailey and Jones, 2019), leading to a growing interest in this domain, with particular recent interest in how to foster and cultivate these critical skills in early childhood and elementary school classrooms. This paper presents results from a randomized evaluation of a low-cost, targeted, classroom-based intervention that uses skill-building games to promote EFs and SR skills in young children. In the following pages, we provide an overview of EFs, links to academic and social–emotional outcomes, and describe the current state of findings from applied interventions designed to build EFs in classroom contexts. We then describe the Brain Games (BG) intervention, our study design and results, and locate the findings in the current body of EF-focused intervention literature.

Given the conceptual confusion related to EFs and SR (Jones et al., 2016; Nigg, 2017), we first operationalize the relevant terms used in this study. We employ SR as an umbrella term for the broad phenomena of children’s regulation (including EFs), or the management and modulation of thoughts, feelings, and behaviors (Karoly, 1993; Jones et al., 2016). As part of the broad SR domain, our conceptualization of EFs follows Bailey and Jones (2019) which presents an integrated model of regulation for school-based interventions. The central idea of this model is that core regulatory processes (i.e., EFs – working memory, response inhibition, and attention control and shifting; Garon et al., 2008) support more complex cognitive, emotion, and social regulation. Combined, these more complex skills form a regulatory gestalt, which more closely reflects how EFs and SR together operate in applied contexts. This model guided our selection of measures because we hypothesize that BGs build core EFs and also shift how children manage in the real world of their classroom environment, acknowledging that this system of regulatory skills are deployed and operate in an integrated way in the real world (Bailey and Jones, 2019). In the following pages, we use the terms executive functions and related self-regulation skills.

### Executive Functions: an Overview

Originating in the cognitive neuroscience literature, researchers generally define EFs as a set of mental processes located in the pre-frontal cortex, or the “control center,” that coordinates thought, memory, emotions, and movement and are used for goal-directed behavior (Miyake et al., 2000; Carlson, 2005; Best and Miller, 2010). EFs (working memory, response inhibition, attention shifting, and attention control) support children and adults to inhibit dominant responses, switch attention between multiple sets, and remember and update information (Miyake et al., 2000; Fuster, 2008), skills required for children to be successful in various settings in which they learn and grow.

For many years, research on EFs was conducted primarily in laboratory-based settings with a focus on examining brain-based processes, or core capacities of the brain. This body of research provided critical knowledge about the presence, malleability, and developmental trajectory of these skills (Garon et al., 2008; Diamond and Lee, 2011). Building on this work and in light of the documented links between EFs, other regulation-related skills, and children’s short- and long-term outcomes (Best et al., 2011; Moffitt et al., 2011), there is a great deal of interest in how young people use EFs when faced with the demands of everyday settings, such as those children face in managing the learning challenges and opportunities of the classroom. For that reason, in addition to defining EFs in academic terms, it is valuable, particularly for applied intervention research, to define and understand EFs in terms of expected classroom behavior. Young children use EFs all of the time, across situations and contexts, and in an increasingly complex and coordinated way over development. For example, in the classroom, working memory plays out in a child’s ability to remember and enact multi-step directions (e.g., when you finish reading, close your folder, wash your hands, and line up at the door), even in the face of distraction, or the ability to remember and build on a previously learned concept or idea. Classroom behaviors tied to attention control and shifting might look like being able to focus attention on a task even in the face of frustration, the ability to move from one task to another (e.g., wrapping up an art project and shifting to math), or thinking about multiple concepts or parts of a problem simultaneously. Finally, response inhibition, or the ability to choose what to pay attention to and what to ignore, might look like staying on task when it is hard or tiring, as well as inhibiting responses and behaviors inappropriate to context or task demands, such as raising one’s hand instead of shouting out the answer.

### Executive Functions and Children’s Outcomes

What emerges from such concrete representations of EFs is that they are inextricably linked to what children need to do in their daily lives, and in particular the basic tasks of learning in the classroom. A large body of research from multiple disciplines indicates that EFs and related self-regulation skills are linked to school readiness and positive adjustment to school, academic achievement, and long-term health and well-being (e.g., Raver, 2002; Eisenberg et al., 2004; Duckworth and Seligman, 2005; Blair and Razza, 2007; Graziano et al., 2007; McClelland et al., 2007; Bull et al., 2008; Best et al., 2011; Moffitt et al., 2011; Valiente et al., 2011).

Focusing on learning outcomes in school, more than a decade of research has established a link between basic executive functions and mathematical skills from preschool through adolescence (Blair and Razza, 2007; Bull et al., 2008; Cragg and Gilmore, 2014; Jacob and Parkinson, 2015; Samuels et al., 2016; Ahmed et al., 2019). Though many studies of EFs focus on links to math achievement, a body of work also demonstrates links to other academic domains including reading (Yeniad et al., 2013) and science (Anthony and Ogg, 2019). A recent meta-analysis of 67 studies including children and youth aged 3–18years found moderate and consistent correlations between EF and math and reading abilities. However, randomized studies of interventions designed to improve EF showed no evidence that gains in EF led to gains in achievement (Jacob and Parkinson, 2015). Thus, existing evidence may support correlational but not necessarily causal links between EFs and academic outcomes (Bailey and Jones, 2019).

Executive functions also play a critical role in children’s abilities to regulate their emotions and behaviors, enabling them to successfully engage in positive social interactions and to manage and resolve social conflicts (e.g., Eisenberg et al., 1995). EFs are linked to a range of social skills, including theory of mind, social competence, empathy, demonstrating socially-appropriate behavior, and social adjustment (Eisenberg et al., 1995, 2004, 2009; Kochanska et al., 1996; Raver et al., 1999; Carlson and Moses, 2001; Sokol et al., 2010). EFs and other SR skills like effortful control (EC) are also linked to lower levels of internalizing and externalizing behaviors, both concurrently and longitudinally, facilitating engagement in social interactions and learning (Kochanska et al., 1996; Eisenberg et al., 2004, 2009).

Research suggests that low-income children and adolescents tend to have lower levels of EFs and emotion and behavior regulation than their more affluent peers (e.g., Noble et al., 2005; Farah et al., 2006; Evans and Kim, 2013; Raver et al., 2013). For example, Noble et al. (2005) report that kindergarteners from low-SES families perform less well on EF tasks than those from middle-SES families and that these differences persist over time (Noble et al., 2007). Exposure to adverse life experiences such as trauma, abuse, neglect, or chronic exposure to other poverty-related stressors are thought to impact the development and functioning of specific regions of the brain responsible for EFs and emotion regulation (e.g., Kishiyama et al., 2008; Bos et al., 2009; Shonkoff and Garner, 2012). Specifically, poverty is associated with poorer selective attention and higher basal levels of the stress hormone cortisol which interrupts use of the prefrontal cortex and the deployment of EFs (Lupien et al., 2001; Kishiyama et al., 2008). However, there is some evidence that regulation-related skills serve as a protective factor for low-income children and youth. For example, in a low-income sample, children and adolescents with stronger EFs and emotion regulation skills were more likely to have positive academic, social/behavioral, and mental health outcomes than children with lower self-regulation, despite similar life experiences (Buckner et al., 2003, 2009).

### Interventions to Promote Executive Functions

EFs can be fostered through repeated exposure to high-quality opportunities to build and practice them. Documented differences along socioeconomic lines may stem from differences in opportunities to practice EF skills (Rosen et al., 2020), and therefore one key lever for their improvement is interventions that provide opportunities to practice EFs in the contexts in which they are relevant and required (e.g., classrooms).

A number of studies have demonstrated that EFs and related SR skills can be improved through direct computer-based training or classroom interventions (Diamond and Lee, 2011; Tominey and McClelland, 2011). In addition, broadly focused, universal, school-based social–emotional learning programs, that often include a focus on EFs and SR, have shown positive impacts on children’s EFs and regulation-related skills, as well as academic and behavioral outcomes (e.g., Riggs et al., 2006; Diamond et al., 2007; Raver et al., 2011).

Efforts to build EFs and SR skills in preschool and school settings tend to happen in two ways: curricular approaches and targeted interventions. Curricular approaches typically involve a series of sequenced lessons implemented over the course of several weeks or months (Jones et al., 2017). Tools of the Mind (ToM), for example, is a curriculum designed to build EFs in preschool settings through imaginative play and self-regulatory language. ToM is based on the Vygotskian concept of “mental tools” and the idea that children’s learning is dictated by their environment until they develop the mental tools to take control of their learning. Prior studies of ToM suggest an overall positive influence on improving children’s self-regulation and decreasing aggression and behavior problems, particularly for sub-groups of students, but also some evidence of mixed and null findings (e.g., Farran and Wilson, 2014; Farran et al., 2015). A recent study of ToM randomly assigned urban daycare sites to the ToM intervention or a pretend play curriculum (the business-as-usual state curriculum in this setting). No main effects of the intervention on children’s executive function measured *via* direct assessment or parent and teacher-reported behavior were found. However, the program was more effective in improving EFs, as measured by the Head-to-Toes (HTT) task, for children who were initially rated by parents as high in hyperactivity/inattention (1 SD above average; ES=0.48; Solomon et al., 2018). In another randomized evaluation of ToM in kindergarten, children assigned to ToM showed improved working memory skills, as measured by the backward digit span (ES=0.14), and improved math skills (ES=0.13), compared to children in the control group. Children in high poverty schools with 75% or more of students eligible for free and reduced-price lunch (15% of the school sample) showed gains in vocabulary (ES=0.43) and reasoning (ES=0.46) and decreased stress physiology (ES not calculated because of complexities with the three-level structure of the data), though this high-poverty sample likely only represents a relatively small number of students (Blair and Raver, 2014). A similar school-randomized study indicated positive and statistically significant impacts of ToM on teacher-reported improvements in self-regulation (ES=0.18), emotion regulation (ES=0.16), and teacher-student relationships (ES=0.15) and decreased behavior problems and aggression (ES=−0.19 for both outcomes) for kindergarten students. Though these findings are promising, it is important to note that the only measurement approach was teacher-reported, which could be subject to bias as teachers were both intervention implementers and raters (Blair et al., 2018).

MindUp, a mindfulness-based curriculum for preschool and elementary aged students, includes core daily deep breathing practice and once weekly lessons and is designed with EF and SR as explicit targets. A randomized trial evaluating MindUp in fourth and fifth grade over a 12-week period found that the program improved response time on three EF tasks (flanker switch trials, incongruent flanker, and hearts and flowers; Cohen’s *d* ranged from −0.21 to −0.31; Schonert-Reichl et al., 2015); however, the trial included only four schools and the data were not analyzed in a manner consistent with the design (i.e., the standard errors were not adjusted for the nesting of students in classrooms and schools). More recently, an evaluation of MindUp in kindergarten classrooms demonstrated reduced executive function deficits, teacher-reported behavioral symptoms and problems, and increased adaptive skills (effect sizes not reported). However, there was no randomization in the study, raising concerns about differences between the two schools that would also produce changes in teacher-reported student outcomes and compounding concerns about teacher-rater bias (Crooks et al., 2020).

While these curricular approaches show promise, due to their comprehensive and often top-down nature, they face a number of barriers (Jones and Bouffard, 2012), including implementation challenges, limited local buy-in, poor integration into educational practice, and low potential for sustainability over time (Jones et al., 2018). These barriers are likely exacerbated in low-income and low-resource contexts. In part to address these challenges, in recent years, researchers are beginning to test the impact of streamlined and more targeted group-based interventions. For example, McClelland and colleagues have developed and tested the Red Light Purple Light (RLPL; McClelland and Tominey, 2015) intervention in three randomized trials with preschool children. In RLPL, children play a series of five music and movement-based games during circle time (for example, having children start and stop moving based on particular cues), with systematic adaptations designed to make the games increasingly challenging over the 16week intervention. In one recent trial, Schmitt et al. (2015) randomized classrooms to either the RLPL intervention, implemented over the course of 8weeks during two short playgroups, or a business-as-usual control group. Children in the intervention group showed statistically significant improvement relative to control students on direct assessments of EFs [Dimensional Change Card Sort (DCCS) and Head Toes Knees and Shoulders (HTKS); Cohen’s *d*=0.16 and 0.32, respectively]. However, there were no changes between treatment and control groups on teacher reported EF skills. It is unclear why there were differential effects for different modes of measurement of the same EF skill (direct assessment vs. teacher report). A potential hypothesis is that the intervention was not long enough for teachers to see meaningful changes in student behavior in the classroom. Additionally, it is possible that teachers are more likely to see changes in EF behaviors when they implement the interventions. In this case, trained facilitators implemented the games, not teachers, and three to five research assistants also participated during each playgroup to support student engagement. Implementation therefore did not reflect a traditional classroom environment. In a more recent study of RLPL, Head Start teachers were assigned to one of three conditions: RLPL intervention, a revised RLPL intervention with added academic content, and a business-as-usual control group. Students’ SR as measured by the HTKS improved (ES=0.31), but the difference between either intervention group and the control group was not statistically significant, perhaps due to insufficient power, given the small sample size (*n*=157 total students; McClelland et al., 2019).

Schmitt et al. (2018) also evaluated the impact of a targeted block play intervention on EF skills in preschool-aged children. Children were randomly assigned to participate in 15-20 minute semi-structured block play sessions two times per week for several weeks or to a business as usual control group. The intervention consisted of small groups of children receiving wooden blocks of various shapes and a short prompt instructing the children to build specific structures (e.g., a tower), which grew more complex over the course of the intervention. The intervention was designed to build EFs and math skills, but there were no statistically significant differences in either for the sample overall. For children with parents with low educational attainment, EFs and math skills improved significantly at post-test (effect sizes not reported). Consistent with Schmitt et al. (2015), a limitation is that the teacher was not involved in delivering the intervention; however, a strength of this approach is that it mimics a concrete classroom practice that can be integrated into everyday classroom teacher practice. Re-structuring block play, a common activity in preschool classrooms, to include increasingly challenging structured prompts may be a relevant and scalable approach that appears to improve key outcomes for sub-groups of children who are likely to struggle.

The body of evidence on interventions designed to cultivate EFs and related SR in school contexts is not definitive. On one hand, there is some promising evidence of the potential for these interventions to improve children’s EFs and SR skills and classroom behaviors. On the other hand, design, methodological, and analytic limitations limit our knowledge of such interventions and their impact on EFs and related SR skills. In this paper, we report the results from a classroom-randomized evaluation of a targeted, game-based intervention designed to improve children’s EFs and SR. The intervention, BG, was designed to address several of the limitations of the interventions summarized above in that they are implemented by teachers, flexible in when and how they are used, and are easily integrated into daily routines. Below, we summarize briefly the evolution of the BG intervention and prior research on its effects.

### Brain Games

#### Brain Games Development: Origins in SECURe

The BG were originally developed and used as a core component of a comprehensive pre-K through fifth grade social–emotional learning curriculum called SECURe (Social, Emotional, and Cognitive Understanding and Regulation in education). In a large pilot study of the effects of SECURe, six schools with 4,000 students in pre-K through third grade classrooms were randomized to either receive the SECURe intervention or to be in the control group (three schools per condition). Results indicated that the program increased students’ attention skills, reduced their impulsive behavior, and had positive effects on literacy skills, especially among the lowest-achieving students in the sample (Jones et al., in preparation). Over 75% of participating teachers reported playing the SECURe BG at least once a week and over a quarter played four or more times a week. In contrast, teachers felt less comfortable with the emotion-focused components of the program and implemented them less than intended. Our working assumption is that the BG, which appealed to teachers due to their targeted nature, simplicity, and flexibility and which were implemented a great deal more than any other components of the program, accounted in large part for the effects we observed in children’s outcomes. We therefore engaged in an iterative design process in collaboration with a graphic design firm, to develop a revised set of stand-alone BG to be implemented separate from the SECURe curriculum.

This new set of BG were piloted in 47 classrooms in three low-income, rural schools in South Carolina over the course of the 2015–2016 school year using a delayed implementation design (*n*=1,248 students). Teachers from two schools were trained in the fall of 2015 and teachers in the third school in the winter (Jones and Imm, 2016). The staggered implementation of BG allowed us to examine the pattern of results across schools with varying implementation schedules. We compared the natural growth of teacher-reported regulation skills with the growth of teacher-reported regulation skills among children who were exposed to BG. In each school, students showed growing levels of self-regulation documented using a teacher-reported measure of children’s EFs and related SR (CEFS, described below), but the average increase was the greatest during periods of exposure to the BG. In addition, at the classroom level, observed classroom practices became more positive (e.g., teachers were employing more positive discipline strategies and were more effectively supporting executive function and SR) over the year, and children in classrooms overall displayed more regulated behavior as the school year progressed (Jones and Imm, 2016).

Across these two early studies, there is a consistent signal suggesting the positive role of BG in improving children’s EFs and related SR and decreasing children’s impulsivity. Importantly, these studies demonstrate that BG have the potential to be a useful strategy to promote these outcomes in lower income and rural settings where teachers are faced with multiple stressors and demands. Overall, we learned that teachers were generally able to integrate BG into their classroom settings and found them easy and fun to do. Building on these positive findings, the primary question this paper addresses is, what is the impact of BG on pre-K through fourth grade children’s executive functions and self-regulation in a low-income urban context? Our previous studies were in lower income rural settings, while this trial takes place in an urban setting with primarily Latinx students. Our decision to evaluate BG in a different setting reflects both our use of a convenience sample and, importantly, our incremental evidence building strategy with which we seek to understand BG in a variety of populations and contexts.

## Materials and Methods

### School Selection and Randomization

The evaluation took place in six schools from a charter school network in a mid-size city serving primarily low-income Latinx students in the northeastern region of the United States. The charter network was recruited through contacts of the principal investigator of the project, and all six eligible schools agreed to participate. Three schools were early childhood centers serving grades pre-kindergarten (pre-K) through first grade and three schools were elementary schools serving children in grades two through four. Each school had two classrooms per grade for a total of six classrooms per school and 36 classrooms total. With our small number of schools overall, in an effort to minimize the impact of differences between them, randomization occurred at the classroom level. Using a random number generator, one classroom in each grade was assigned to the intervention group and one classroom to the business-as-usual control group for a total of 18 classrooms in each condition. Teachers in the intervention group participated in BG training and implemented the intervention (described below). Teachers in the control condition continued with business-as-usual classroom activities.

### Participants

Participants included 626 children (50.64% boys) in pre-K through fourth grade. Children were 95.43% Latinx, 3.11% White, and <1% Black/African-American and Other. As noted above, classrooms were randomly assigned within each school to the BG intervention group (*n*=322) or to a business-as-usual control group (*n*=304). Following randomization, independent sample *t* tests indicated that the children did not differ significantly across study conditions on baseline demographic characteristics, suggesting that the randomization process was successful. The sample also included 65 teachers (99.98% female, average age=30.25years, *SD*=7.78). Approximately 63% of teachers identified as White, 3% as Black or African American, 12.5% as Hispanic or Latino, less than 1% as Asian, and 20.3% as Other. On average, teachers had worked in the profession for 6.34years, *SD*=3.55 and in the current school for 3.53, *SD*=2.34. Independent sample *t* tests indicated that there were no statistically significant differences between treatment and control teachers on the number of years teachers had worked in the profession, years in the current school, or age.

Aggregated data from students in our sample indicate that an average of 11.64% of students were classified as special education, ranging from 7.06 to 16.67% in each school and from 0 to 33.33% across all classrooms. Around 38.18% of students in our sample across all schools and classrooms were classified as English Language Learners, ranging from 22.52 to 66.32% in each school and from 4.45 to 90% across all classrooms. Publicly available online data indicate average levels of students classified as economically disadvantaged [based on a student’s participation in state-administered programs, e.g., Supplemental Nutrition Assistance Program (SNAP)] range from 60.3 to 64.8%, however these numbers reflect an average from all students in grades pre-K through eight; data are not available only for the grades in our sample.

### Missing Data

**Figure 1:**
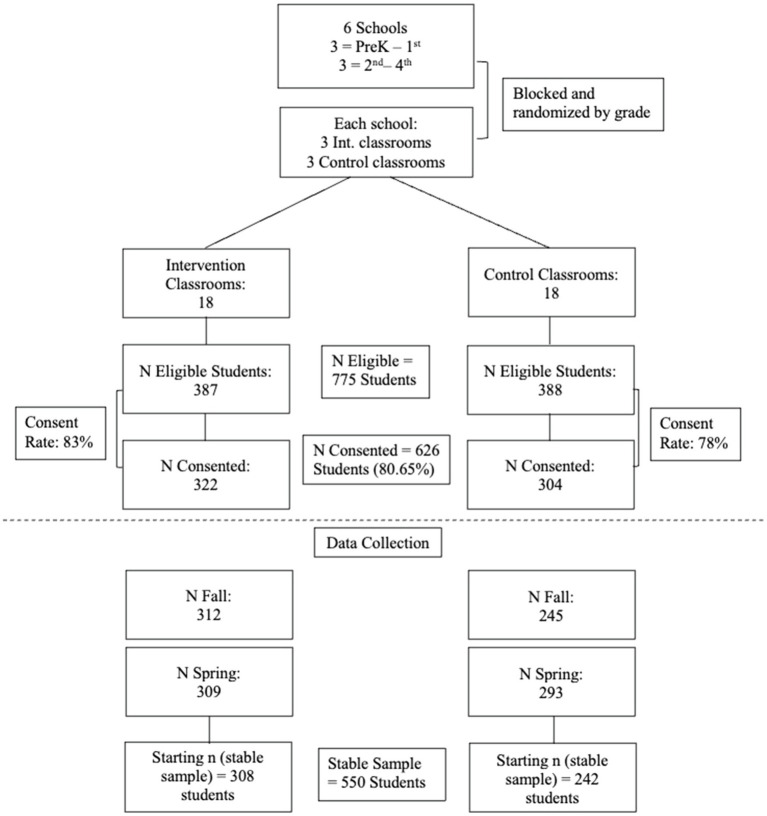
Sample breakdown.

Challenges with data collection led to variability in sample sizes between waves. All analyses were conducted using only the stable sample, meaning children for whom we have data at both time points. See Figure 1 for a breakdown of eligible and consented students as well as the final stable sample used in all analyses. To examine whether the stable sample differed systematically from the full sample, unpaired sample *t* tests were used to compare the stable sample to all children in the fall and then to all children in the spring for the treatment and control groups separately for all outcomes as well as for the child-level covariates. The *t* tests were not statistically significant for any variables except two outcomes. In the control group, the mean of the stable sample for teacher-reported prosocial behavior was statistically significantly higher in spring than the mean of the full sample. In both treatment and control groups, teacher-reported teacher-student relationship quality in spring were statistically significantly lower in the stable sample than the full sample.

### Procedure

Data were collected during the fall and spring of the 2017–2018 school year. In the beginning of the school year, consent forms in English and Spanish were sent home to all families informing them of the study and seeking consent for their child to participate in data collection. The overall consent rate was 80.65% across grades (ranging from 65.65% in the first grade to 91.34% in the fourth grade) and consent rates did not differ significantly between the intervention (82.94%) and control (78.35%) groups.

Information on student skills was collected through direct assessments conducted by trained assessors and teacher reports. Assessors participated in an in-depth 1-day training with members of the research team to learn about and practice administering the assessments. The assessors, who were blind to treatment conditions, then visited each school to administer the one-on-one direct assessments. In two schools (one early childhood center and one lower school), nearly all students with consent participated in the direct assessment. Due to substantial constraints on time for direct assessments, a randomly selected group of six consented children (three male, three female) from each classroom were chosen to participate in the direct assessment in four of the six schools, representing approximately 30% of the students in each classroom. The sampling of children within classrooms to address school limits on data collection time follows established practice for RCTs (e.g., Morris et al., 2014). Prior to beginning the assessment, children learned about the study and activities and provided verbal assent. The assessment took approximately 5–10min for pre-K and kindergarten students and 30min for first through fourth grade students. Children identified by their teacher as primarily a Spanish speaker received an assessment translated into Spanish and were assessed by a Spanish speaking assessor. Only a handful of students (three students in pre-K and one in first grade) received assessments in Spanish, and we expect, given the very small number, that this likely did not have an impact on outcomes. After administering the assessment, the assessor completed a short survey about the child’s behavior during the assessment period.

At each timepoint, teachers completed reports about each student in their classroom as well as the characteristics of their classroom. In pre-K through second grade classrooms, there were two teachers in each classroom. In the third and fourth grade, there was one teacher per classroom and one teacher who floated between the two classrooms at each grade level. In the younger grades, surveys were distributed to both teachers in the classroom. Floater teachers received a survey to complete for a randomly selected subgroup of students from all of the classrooms with which they work. The paper-based surveys were distributed in-person to each school and were collected approximately 1–2weeks later. The same data collection procedures were conducted in fall and spring of the 2017–2018 school year.

### Intervention

Brain Games is a classroom-based intervention designed to build preschool and elementary-aged children’s EFs and related self-regulation skills. The intervention consists of a small box of 31 games, each designed to target one of three core EF skills, called Brain Powers: attention control (Focus Power), working memory (Remember Power), and inhibitory control (Stop and Think Power). For example, in the “Remember Power” game titled “On My Pizza, I Like” students are asked to add pizza toppings to a pretend pizza and to remember the increasing list of pizza toppings – targeting working memory by requiring students to remember and manipulate information in their minds. The card provides prompts to make the games both simpler or more challenging (e.g., substituting pizza toppings for math concepts or vocabulary words, adding topics in alphabetical order). After each game, teachers are encouraged to lead discussions to help students recognize the skills they are building, identify successful strategies, and connect skills to other times of the day when they need to focus, remember, or use self-control. In “On My Pizza, I Like,” the post-game talk might include a discussion of how students use their Remember Brain Power to keep track of the toppings in the right order (e.g., repeat the ingredients to myself, create a picture in my mind) and how those strategies can apply to other times of the day when Remember Power is needed (e.g., packing school bag in the morning). Brain Games are not implemented during a designated period (as most SEL interventions are), rather teachers are encouraged to integrate the games into their daily routines and transitions across classroom activities and various settings (classroom, hallways, recess, etc.). The box also includes concrete strategies and support for teachers; key findings about brain development, learning, and behavior; and a set of three classroom posters to help reinforce the use of skills throughout the day. Prior to the beginning of the school year, intervention teachers participated in a 90-min BG training that covered the science behind the development of EFs and self-regulation, introduced the games, and provided teachers with scaffolded opportunities to practice. In May, teachers in the intervention group received four virtual booster training modules focused on various implementation topics (e.g., plan for integrating BG and academic content, improving implementation using BG best practices).

The BG Theory of Change (ToC) is closely aligned to the BG intervention and the three “Brain Powers.” The expected near-transfer outcomes include (1) the core EF skills of attention control, working memory, and inhibition and (2) classroom tone and relationships (teacher–student and peer relationships), which the games foster through fun and inclusive game play as well as rich post-game talk. Medium-transfer outcomes include prosocial behavior, impulse control, and broader behavioral regulation and positive behavior, which build on the foundational EFs and SR skills, and fewer disciplinary events. Importantly, the ToC guided the selection of measures used in the study, which are closely related to the key components and goals of the BG. This paper focuses only on child-level outcomes.

### Measures

The primary constructs and measures are presented below by measurement type, followed by the child and classroom baseline covariates.

#### Direct Assessments of EFs and Related Self-Regulation Skills

Our battery of EF direct assessments was designed to align with the BG ToC and to capture component EF skills as well as more global EFs. Two assessments were used to assess EFs with pre-K and kindergarten children, Pencil Tap and DCCS. The DCCS, Corsi Blocks (Forward and Backward Digit Spans which capture short-term and working memory), and Trail-Making-Task were used with first grade through fourth grade children. After each assessment, the trained assessor completed the Preschool Self-Regulation Assessment Assessor Report (PSRA-AR).

Inhibitory control was assessed in pre-K and kindergarten children using the Pencil Tap. In this task, the assessor tapped a pencil either once or twice and the child was instructed to tap the opposite number of times (i.e., correct response of one tap would be two taps, correct response of two taps would be one tap). Because children in the early years (e.g., pre-K vs. kindergarten) vary so much in motor capacity, the total time to complete all 16 trials was recorded and then controlled in all analyses (see Analytic Plan below). Scores were created by calculating the mean number of correct responses (if less than 80% were missing) and dividing by the total number of trials. If more than 80% of responses were missing for a child, a total score was not calculated for the Pencil Tap. Children who were correct more often received higher scores.

Global EF was assessed using (1) the DCCS for all children in the sample and (2) the Trail Making Task (TMT) for first through fourth graders. The DCCS card-sorting task (Zelazo, 2006) was used with all children across grades to capture global EF. The task consists of three phases. In the first phase (the pre-switch phase), the child sorted blue and red cards by color. In the second phase (the post-switch phase), the child sorted the same cards by shape (boat or rabbit). If the child successfully completed the pre- and post-switch phases, they participated in the border phase. In this phase, children are instructed to sort the cards by color if the card has a border and by shape if the card does not have a border. The child must decide how to sort (color or shape) based on whether or not the card had a border. Because we used a paper and pencil version, only accuracy was recorded. Scoring followed the protocol outlined by Zelazo (2006). Children received a zero if they failed the pre-switch phase, one point if they passed the pre-switch phase but did not pass the post-switch phase, two points if they passed the pre- and post-switch phases but not the border phase, and a three if they passed the border phase by getting at least nine of the 12 border cards correct. Children who were able to accurately sort the cards based on the rules of the phase were given higher scores.^1^

This task has been described by researchers as a measure of attention shifting, working memory, cognitive flexibility, and as an index of self-control/complex EF (e.g., McClelland et al., 2014; Karalunas et al., 2016; Bailey et al., 2018). Based on our analysis of the task and the behavior observed, we categorize or describe this task as a measure of global EF.^2^ The phases of the DCCS tap multiple sub-components of EF. In particular, the border version requires inhibition (inhibiting the impulse to sort immediately), working memory (keeping the rule structure in mind), and attention shifting (shifting based on border/no border and other dimensions; Bailey et al., 2018).

The TMT is primarily used as a measure of attention control, attention shifting, and global EF. The TMT has three parts. In the first part, children are asked to draw a continuous line connecting numbers in sequential order. In the second part, children draw a line connecting letters. In the third part, children connect numbers and letters in alternating order (i.e., 1-A-2-B, etc.). Before each part, each child completed a short trial to ensure they understood the directions. The number of parts completed and total completion time were recorded. Children received zero points if they did not complete any of the parts, one point if they completed either Part 1 (numbers) or Part 2 (letters), two points if they completed both 1 and 2, and three points if they completed Part 1, Part 2, and Part 3 (mixed numbers and letters). Children who complete more parts have higher scores. As with the Pencil Tap, due to developmental variation in motor skills, the total completion time is used as a covariate in all analyses.

Short-term memory and working memory were assessed using the Forward Digit Span (FDS) and Backward Digit Span (BDS), respectively, of The Corsi Blocks. In the first phase of this task, the experimenter tapped a series of blocks and asked the child to tap the blocks in the same order (FDS). In the second phase, the experimenter tapped a series of blocks and asked the child to tap the blocks in the reverse order (BDS). The task starts with only two blocks, and another block is added each time the child taps a pattern correctly. The process continues up to a maximum of nine blocks for forward digit span and six blocks for the backward digit span. The child completed two test trials (each beginning with two blocks) for each phase (forward and backward). The highest number of blocks tapped correctly was recorded and averaged across both trials in each phase (i.e., across the two forward trials) to generate the final scores. Children who correctly recalled a greater number of blocks received higher scores.

Attention and impulsivity was measured using the Attention and Impulsivity subscale of the Preschool Self-Regulation Assessment – Assessor Report (PSRA-AR; Smith-Donald et al., 2007). The assessor completed this 12-item report of children’s attentional, behavioral, and emotional regulation after the child completed the direct assessment. We did not include four additional items sometimes included in the Attention/Impulsivity scale given extremely low variability in these items (i.e., 96% of the sample was rated as not defiant and 99% were rated as not aggressive). For each item, assessors responded on a Likert scale (0–3) rating the child’s behavior during the direct assessments. Sample items include “Careful, interested in accuracy; not careless” and “Child has difficulty waiting between tasks.” Cronbach alphas for this scale across the two waves range from 0.87 to 0.89. High ratings indicate higher levels of attention and lower levels of impulsivity during the assessment.

#### Teacher Survey of Classroom Behavior

In fall, 37% of the teacher surveys across intervention and control groups were completed by two teachers. In spring, 60% of the surveys were completed by two teachers. Surveys were highly correlated between fall and spring and between the two teachers (>0.7 for all scales) and were therefore combined to create one teacher rating per timepoint (i.e., fall or spring) per child. All scale scores were computed as the mean across the items for each construct. Scores were only calculated if fewer than 80% of items were missing for each scale.

Regulation-related behavior was measured using teacher reports on the Classroom Executive Function Survey (CEFS; Jones S. et al., 2015), a measure designed to capture the behavioral manifestations of children’s executive functions and SR skills in classroom settings. The survey contains 12 items that ask teachers how often they have observed the child engaging in specific behaviors such as “following multiple step instructions,” “waiting to be called on before responding,” and “becoming easily distracted” in the preceding 3weeks. Items are rated on a five-point scale (*never*=0; *all of the time*=4). Cronbach alphas for this scale for fall and spring are 0.95–0.97, respectively. Higher ratings on the CEFS indicate higher levels of EFs and related self-regulation skills.

Prosocial behavior was measured using items adapted from the prosocial behavior scale of the Teacher Observation of Classroom Adaption-Revised (TOCA-R; Werthamer-Larsson et al., 1991; Koth et al., 2009). The survey contains seven items that ask teachers how often a set of statements was true about a child, including “had many friends,” “teased classmates,” and “showed empathy and compassion for others’ feelings.” Cronbach’s alpha for fall and spring are 0.87 and 0.89, respectively. Higher teacher-reported scores on the TOCA-R indicate higher levels of prosocial behavior.

The teacher–student relationship was measured using an adapted version of the Teacher Student Relationship Scale – Short Form (TSRS-SF; Pianta, 2001). The survey contains seven items from the conflict scale (e.g., “dealing with this child drains my energy” and “this child and I always seem to be struggling with each other”) and three items from the closeness scale (e.g., “this child openly shares his/her feelings and experiences with me”) and asks teachers to rate the degree to which the statement currently applies to their relationship with the child. Items are rated on a five-point scale (*definitely does not apply*=0; *definitely applies*=4). Items were combined across scales to create an average relationship score. Cronbach alphas for fall and spring are 0.89 and 0.90, respectively. Higher scores on the TSRS indicate a closer, less conflictual teacher–student relationship.

Discipline events were measured using a set of four items that ask the teacher how often the student had been sent to the principal’s office, to an in-school suspension, home or removed from school, or to an alternative classroom in the preceding 3weeks. Items are rated on a three-point scale (*none*=0; *two or more times*=2). Items were averaged to create a final discipline score, with higher scores indicating more frequent discipline. Cronbach’s alpha was not calculated for discipline events because it is a count.

#### Baseline Covariates

Classroom Characteristics were measured using 10 items designed to assess teachers’ perceptions of their classroom adapted from the Classroom Appraisal of Resources and Demands (CARD; Lambert et al., 2001). Teachers were asked to provide a numerical answer to questions about approximately how many children “come from homes in which English is not the primary language,” “have behavior problems,” and “are performing below grade level.” Teachers also responded to a set of demographic questions asking them to report gender, race/ethnicity, and number of years at the current school. Teacher demographic characteristics are described above but were not used as covariates in any analyses (see Analytic Plan below for more details).

In addition to classroom characteristics, child gender, English language learner status (ELL), and special education status (SPED) were compiled from administrative records and included as baseline covariates.

### Analytic Plan

We began by conducting a set of descriptive analyses including an examination of attrition (overall and by treatment status) and baseline equivalence. We examined overall attrition in two ways. First by comparing the number of students who consented to participate in the study to those who remain and for whom we have spring outcome data and second by comparing the number of students for whom we have fall data to those who remain and for whom we have spring outcome data. We also examined differential attrition, or attrition by intervention status, using both approaches. Differences between children who attritted vs. those who did not were calculated by regressing a dummy variable for attrition (1=attritted) on child demographic characteristics with school fixed effects and classroom random intercepts included. A significant coefficient would indicate a significant difference between children who attritted and those who did not on that variable.

To assess baseline equivalence, following What Works Clearinghouse Guidelines (WWC), a standardized mean difference or effect size was calculated for each continuous variable using Hedge’s *g* (dividing the difference between the adjusted means for the treatment and control groups by the pooled, unadjusted SD) and each dichotomous variable (e.g., gender) using Cox’s Index.

To examine the average impact of BG on children’s outcomes, we used an intent-to-treat (ITT) approach. As described in the Materials and Methods section, randomization occurred at the classroom level such that children were nested in classrooms and classrooms were nested in schools. To account for the nesting of students in classrooms, we employed multilevel models with random intercepts for each classroom. All models also included school fixed effects and controlled the relevant outcome measures at baseline. Each outcome was included in a separate model. For outcomes with both time and accuracy scores (Pencil Tap and TMT), time was included as a covariate. To determine the significance of effects, we adhered to traditional significance levels of *p*<0.05 and also note marginal significance at *p*<0.10. Effect sizes are also provided for all statistically significant outcomes which were calculated by dividing the relevant treatment estimate by the SD of the control group (a variant of Cohen’s d, called Glass’s ∆, referred to in the results as ES; Glass et al., 1981; Cohen, 1992; Jones et al., 2010).

In the following section, we present results from a taxonomy of four models for each of our primary outcomes with sets of covariates added across models: (1) no covariates, (2) only child-level covariates including gender, ELL status, and SPED status, (3) only classroom covariates including teacher reported number of children (i) in the classroom, (ii) from homes in which English is not the primary language, (iii) developmentally behind most of the other children, (iv) with learning disabilities, (v) who have poor attendance, (vi) who have behavior problems, and (vii) who are performing below grade level, and (4) combined child and classroom covariates. We interpret Model 2, with child-level covariates, and Model 4, which includes both child- and classroom-level covariates due to (1) the association between classroom covariates and implementation (described on pp. 31 and 32 below), (2) associations between classroom covariates and child outcomes (see Table 1), and (3) differences in baseline classroom characteristics between the intervention and control groups (see Table 2). Model 4 employs a smaller sample than does Model 2 due to missing data, but as we note below, these two samples do not differ significantly on child demographic characteristics or on baseline child outcomes.

**Table 1:** Correlation matrix of classroom characteristics and child outcomes.

	Inhibitory control (PT)	Global EF (DCCS)	Global EF (TMT)	Short term memory (FDS)	Working memory (BDS)	Attention/Impulsivity (PSRA-AR)	Regulation-related skills (CEFS)	Prosocial behavior (TOCA)	Teacher–student relationship (TSRS)	Discipline events
Number of children who are in the classroom	0.17	−0.15^*^	−0.23^*^	−0.16^*^	−0.19^*^	−0.05	−0.06	−0.09+	−0.02	−0.03
Number of children who come from homes in which English is not the primary language	0.25+	−0.12+	−0.08	−0.03	−0.03	−0.15^*^	0.05	−0.01	0.05	−0.02
Number of children who are developmentally behind most of the other children	−0.13	−0.07	−0.07	−0.14+	−0.04	−0.17^**^	−0.09+	0.03	−0.02	0.02
Number of children who have learning disabilities	0.03	0.03	0.03	−0.06	0.00	0.02	−0.08	0.01	−0.08+	−0.03
Number of children who are gifted or talented	−0.12	0.09	−0.06	0.00	0.02	−0.18^**^	0.01	0.02	0.03	0.13^*^
Number of children who have poor attendance	0.14	−0.13^*^	−0.23^***^	0.06	−0.05	−0.03	−0.04	−0.14^**^	−0.06	−0.0531
Number of children who have behavior problems	−0.11	−0.13^*^	−0.11	−0.06	0.04	−0.15^*^	−0.13^**^	−0.03	−0.21^***^	0.18^***^
Number of children who are performing below grade level	0.03	0.01	0.15^*^	0.13+	0.23^***^	0.15^**^	−0.01	0.15^**^	−0.08	0.08+

PT, Pencil Tap; DCCS, Dimensional Change Card Sort; TMT, Trail Making Task; FDS, Forward Digit Span; BDS, Backward Digit Span; PSRA-AR, Preschool Self-Regulation Assessment – Assessor Report; CEFS, Child Executive Function Survey; TOCA, Teacher Observation of Classroom Adaptation; and TSRS, Teacher Student Relationship Scale.

* *p*<0.05;

** *p*<0.01 and

*** *p*<0.001.

**Table 2:** Descriptive information on baseline classroom characteristics.

	Full sample			Intervention group				Control group				Stand. mean dif.
	*N*	Unadjusted mean	Pooled SD	*N*	Mean	Adj. mean	SD	*N*	Mean	Adj. mean	SD	
Number of children who are in the classroom	27	21.33	1.66	15	21.47	20.33	1.81	12	21.17	20.51	1.53	0.11
Number of children who come from homes in which English is not the primary language	27	13.91	5.92	15	13.03	12.57	6.37	12	15.00	14.90	5.36	0.39
Number of children who are developmentally behind most of the other children	27	2.16	1.48	15	2.52	3.52	1.36	12	1.71	2.97	1.54	0.37
Number of children who have learning disabilities	27	2.02	1.27	15	1.77	1.33	1.29	12	2.33	2.01	1.21	0.54
Number of children who have physical disabilities	27	0.07	0.27	15	0.07	0.19	0.26	12	0.08	0.21	0.29	0.06
Number of children who are gifted or talented	26	0.60	1.22	15	0.80	1.18	1.47	11	0.32	0.73	0.72	0.37
Number of children who are homeless or transient	25	0.04	0.20	15	0.00	0.15	0.00	10	0.10	0.27	0.32	0.56
Number of children who have poor attendance	27	0.57	1.01	15	0.37	0.58	0.81	12	0.83	1.13	1.19	0.54
Number of children who have behavior problems	27	3.00	1.73	15	3.03	4.03	1.96	12	2.96	4.21	1.48	0.11
Number of children who are performing below grade level	27	5.04	2.47	15	5.10	5.31	3.06	12	4.96	5.29	1.57	0.01

*F*-test for overall balance revealed no statistically significant difference between classrooms assigned to intervention vs. control groups (*F* (15, 9)=0.65, *p*=0.7773). *F*-tests included school fixed effects.

Overall, our study is underpowered, which influences our interpretation of null findings as we note in the Discussion. Specifically, assuming an alpha of 0.05 and power of 0.80, our total possible sample size of 775 is sufficient to detect minimum mean differences of ~0.31 *SD* between treatment conditions, and the stable sample of 550 is sufficient to detect minimum mean differences of ~0.37 *SD* between treatment conditions. Our ability to detect effects are further reduced for the direct assessments, which include both fewer students and, in many cases, fewer classrooms (i.e., direct assessments were conducted only in particular grades).

## Results

### Preliminary Analyses

Figure 1 summarizes eligible and consented students as well as the final stable sample used in all analyses. Seven hundred and seventy-five students were eligible, 626 consented to participate, 557 participated in fall data collection, 602 participated in spring data collection, and 550 students across treatment and control groups participated in both. Descriptive statistics for child level demographics and classroom characteristics are presented in Tables 2 and 3 and descriptive statistics for the primary child outcomes for the full analytic sample as well as for intervention and control groups are presented in Table 4. Adjusted treatment and control group means in Table 4 were calculated by regressing treatment status on each pre-test score and salient demographic characteristic. All models were adjusted to account for the study design and include school fixed effects and classroom random intercepts.

**Table 3:** Sample baseline demographic characteristics.

Child-level	Intervention group (*n*=308)	Control group (*n*=242)	Total (*N*=550)
Child grade			
Pre-kindergarten	48	32	80
Kindergarten	45	47	92
First grade	42	30	72
Second grade	56	55	111
Third grade	57	41	98
Fourth grade	60	37	97
Child gender			
Boys (%)	48.69	53.11	50.64
Girls (%)	51.31	46.89	49.36
Child race/ethnicity			
White (%)	3.27	2.90	3.11
Black/African–American (%)	<1	<1	<1
Hispanic/Latino (%)	95.10	95.85	95.43
Other (%)	<1	<1	<1	Other child characteristics
English language-learner (%)	37.34	39.26	38.18
Special-education classification (%)	11.04	12.40	11.64

**Table 4:** Descriptive information on all outcomes and baseline equivalence.

	Full sample			Intervention group				Control group				Stand. mean dif.
	*N*	Unadjusted mean	Pooled SD	*N*	Mean	Adj. mean	SD	*N*	Mean	Adj. mean	SD	
Direct assessments of children’s EF and related self-regulation skills												
Pre-K and kindergarten												
Inhibitory control (Pencil Tap)	75	0.77	0.27	39	0.74	0.74	0.30	36	0.81	0.81	0.24	0.28
Grades 1–4												
Global EF (Trail Making Task)	227	2.75	0.57	111	2.77	2.28	0.56	116	2.73	2.32	0.60	0.06
Short term memory (Forward Digit Span)	230	4.50	0.99	116	4.56	4.04	0.97	114	4.45	3.97	1.01	0.07
Working memory (Backward Digit Span)	230	3.92	1.12	116	3.98	3.31	0.96	114	3.86	3.27	1.26	0.04
All students												
Global EF (Dimensional Change Card Sort)	330	2.11	0.73	167	2.13	1.78	0.76	163	2.10	1.76	0.70	0.03
Attention/Impulsivity (Assessor Report)	334	2.57	0.45	166	2.57	2.37	0.46	168	2.56	2.36	0.44	0.02
Teacher survey of classroom behavior												
Regulation-related skills (CEFS)	465	2.78	0.87	274	2.77	2.58	0.86	163	2.10	1.76	0.70	0.03
Prosocial behavior (TOCA)	465	4.28	0.72	274	4.33	4.43	0.77	191	4.20	4.32	0.64	0.15
Teacher-student Relationship (TSRS)	453	3.33	0.79	274	3.34	3.29	0.84	179	3.32	3.29	0.72	0.02
Discipline events	483	0.08	0.23	274	0.09	0.11	0.25	209	0.06	0.08	0.19	0.16
Demographic characteristics												
Gender (female=1)	547	0.49	0.50	306	0.51	0.49	0.50	241	0.47	0.48	0.50	0.10
SPED	550	0.12	0.32	308	0.11	0.11	0.31	242	0.12	0.14	0.33	0.07
ELL status	550	0.38	0.49	308	0.37	0.37	0.48	242	0.39	0.39	0.49	0.04

*F*-test for overall balance on the pre-K and K measure of inhibitory control (Pencil Tap) revealed no statistically significant difference between students assigned to intervention vs. control groups (*F* (3, 11)=0.61, *p*=0.6237). *F*-test for overall balance on direct assessments in Grades 1–4 revealed no statistically significant difference between groups assigned to intervention vs. control groups (*F* (8, 23)=0.06, *p*=0.9998). *F*-test for overall balance on direct assessments used in all grades revealed no statistically significant difference between students assigned to intervention vs. control groups (*F* (7, 35)=0.00, *p*=1.00). *F*-test for overall balance on initial balance in the teacher survey of classroom behavior revealed no statistically significant difference between groups assigned to intervention vs. control groups (*F* (9, 27)=1.03, *p*=0.4413). *F*-tests included school fixed effects and clustered standard errors at the classroom level.

To examine attrition, we first compared students who consented to participate in the study (626 students) with those who have data for at least one outcome at the end of the study (602 students). Approximately 3.83 percent of the children who consented to participate in the study at baseline did not participate in data collection at the end of the study. Rates of attrition for the treatment and control groups were similar at 4.04 and 3.62%, respectively. With overall levels of attrition at 3.83 percent and differential attrition at less than 1%, the study falls into acceptable rates of attrition according to WWC under the conservative attrition standards (What Works Clearinghouse, 2017). We note that the stable sample has 550 students, which is fewer than the 602 students with spring outcome data, because fewer students participated in baseline data collection (557 students). Second, we examined attrition by looking at the number of students for whom we have both baseline fall data and spring outcome data. Five hundred fifty-seven of the 626 consented students participated in baseline data collection. Of those 557, only seven students (1.26 percent) did not participate in data collection at the end of the study (four from the intervention group and three from the control group, or approximately 1% from each group), which is also acceptable under conservative attrition standards. Students who attritted from the sample were significantly more likely to be female (six of seven were female). There were no other significant differences based on ELL or SPED classifications. Differences between attritted and non-attritted students were not calculated on the measure of global EF (TMT), short term memory (FDS) or working memory (BDS), as only one student with baseline data did not have spring outcome data.

Results of analyses examining baseline equivalence across intervention and control conditions in child demographic characteristics and baseline outcomes for the sample used in Model 2 are shown in Table 4. The three child demographic characteristics (gender, SPED, and ELL status) and eight of the nine baseline outcomes had a standardized mean difference (SMD) below 0.25, indicating small differences between intervention and control groups that can be satisfied by statistical adjustment. Only the measure of inhibitory control used in pre-K and kindergarten (Pencil Tap) showed a SMD greater than 0.25 (SMD=0.28), where children in the intervention group scored lower than children in the control group, on average. All baseline equivalence findings between the treatment and control groups were consistent in the smaller sample used in Model 4 (see Appendix). The samples from Models 2 and 4 do not differ significantly from each other on child demographic characteristics or on baseline child outcomes.

Examination of baseline classroom characteristics reveal standardized mean differences between intervention and control groups requiring covariate adjustment (see Table 2). Specifically, teachers in the intervention group reported having more students considered developmentally behind their peers, while teachers in the control group rated their classrooms as having more students in their classrooms, coming from homes in which English is not the primary language, with learning disabilities, behavior problems, and poor attendance. In addition to the statistical differences in classroom covariates between the groups, we consider these classroom characteristics to have *practical* significance in that they can influence implementation of BG and therefore their impacts on outcomes, as well as overall classroom functioning. For example, in larger classrooms, gaining and maintaining student attention and engagement may be more challenging than in classrooms with fewer students. In addition, as is the case with this study, in larger classrooms with more students who are considered to be developmentally behind their peers, the teacher may need to either provide various adaptations for students or provide additional support to students who are struggling. We also note that the *F*-test for overall balance revealed no statistically significant differences between classrooms assigned to intervention vs. control groups *F* (15, 9)=0.65, *p*=0.78.

### Implementation of Brain Games

Implementation of Brain Games was systematically tracked during the 40-week implementation period (end of August through mid-June, accounting for school breaks). Teachers completed weekly log-books in which they tracked how many games they played and the number of minutes per game. Teachers were asked to play one game per day, or five games per week. On average, teachers played three games per week (*SD*=1.26) for an average of 14 total minutes per week (*SD*=6.74). Pre-K classrooms tended to play the most games, approximately four per week (*SD*=0.16) and second grade classrooms spent the most time playing BG each week, playing for an average of 21min weekly (*SD*=7.09). We also note that Brain Game play was high in the older grades, with third and fourth grade classrooms playing an average of 3.73 (*SD*=2.17) and 1.79 (*SD*=0.27) games per week, respectively.

If BGs were implemented fully as intended, children would have played 200 total games over the course of the 40-week implementation period (five games per week or one per day). Average implementation at the grade level ranged from approximately 72 in fourth grade (*SD*=10.69) to 157 games in pre-K (*SD*=6.43). Notably, only one third grade classroom played the intended number of games per week, suggesting that more work is needed to understand the feasibility of daily implementation and thresholds for implementation effectiveness (i.e., how much game play is needed to generate intervention impacts).

Correlational analysis examining associations between implementation and baseline classroom covariates indicate that teachers were significantly more likely to implement BGs in classrooms rated as having greater numbers of students in them (*r*=0.36), students coming from homes in which English is not the primary language (*r*=0.27), students with learning (*r*=0.14) or physical disabilities (*r*=0.63), and students who have poor attendance (*r*=0.27). Teachers were significantly less likely to play BGs in classrooms with higher numbers of children performing below grade level (*r*=−0.27). These associations suggest an important link between how teacher view their classrooms and their interaction with and use of the intervention.

We do not have data from the current study on the exact times in the day teachers chose to play the games, and what BG may have replaced in intervention classrooms since teachers had full discretion as to when and how to play. Implementation data from a past study of BGs in South Carolina suggest that BGs might make up for some time otherwise lost to transitions or wait time (e.g., waiting in line to enter the cafeteria or whole class trips to the bathroom after lunch), fit well as students arrive or during morning meeting, and could help students refocus, calm down, and transition from one activity to another. In addition, teachers integrate BGs into academic content (e.g., playing Simon Says with math angles or I Spy with letter sounds), blending BGs with core academic content. Unlike interventions that replace a part of the day such as RLPL which took place over 16 structured playgroups (length of playgroups not reported; Tominey and McClelland, 2011), or MindUP which is implemented once a week for 12weeks for 40–50min, BGs afford flexibility in length of time devoted to the games and when the games are implemented so they do not necessarily replace critical classroom activities.

### Impacts on Child Outcomes

#### Direct Assessments of EF and Related Self-Regulation Skills

Models 1 and 2 of Table 5 present the impact of BG without covariates and controlling child demographic covariates, respectively. In Model 2, with child covariates, there was no effect of the BGs on the direct assessments of inhibitory control (Pencil Tap), global EF measured with the Trail Making Task, short term memory, or working memory. We note that in the Pencil Tap, nearly 70% of the sample in the fall and 94% of the sample in the spring scored at least 80% correct, indicating a ceiling effect. There was a statistically significant negative effect of the BG on global EF as measured by the DCCS (*b*=−0.15, *S.E.* =0.07, *p*<0.05); however, we note that this effect was not robust to alternative approaches to scoring the DCCS. A continuous scoring approach (e.g., McClelland et al., 2014) where the number of correct cards across all rounds were summed was not statistically significant (*b*=−0.66, *S.E.*=0.50, *p*=0.12). There was a marginally significant effect on the attention/impulsivity subscale of the Assessor Report (*b*=0.07, *S.E.* =0.04, *p<*0.10). In Model 4, in which both child and classroom covariates were included, we did not observe statistically significant impacts of BG on the direct assessments of inhibitory control (Pencil Tap), short-term or working memory (Corsi Blocks), or global EF measured *via* the DCCS. There was, however, a statistically significant effect on global EF measured by the Trail Making Task, *b*=0.23, *S.E.* =0.11, *p*<0.05, with an effect size of nearly 1/2 a SD (ES=0.41). In addition, we observed impacts on attention/impulsivity measured using the PSRA-Assessor Report, *b*=0.11, *S.E.* =0.05, *p*<0.05, with an effect size of nearly 1/3 of a SD (ES=0.27).

**Table 5:** Impact of brain games (BG) on children’s outcomes.

	Model 1: no covariates	Model 2: child covariates	Model 3: classroom covariates	Model 4: child and classroom covariates
Direct assessments of children’s EF and related self-regulation skills				
Pre-K and kindergarten				
Inhibitory control (PT)	−0.02 (0.02) 75	−0.03 (0.02) 75	0.02 (0.11) 47	0.0.02 (0.11) 47
Grades 1–4				
Global EF (TMT)	−0.03 (0.06) 227	−0.03 (0.06) 227	0.24^*^ (0.11) 172	0.23^*^ (0.11) 172
Short term memory (FDS)	−0.16 (0.11) 230	−0.16 (0.10) 230	0.05 (0.20) 174	0.07 (0.20) 174
Working memory (BDS)	0.03 (0.11) 230	0.03 (0.11) 230	−0.03 (0.22) 174	−0.03 (0.22) 174
All students				
Global EF (DCCS)	−0.15^*^ (0.07) 330	−0.15^*^ (0.07) 330	−0.09 (0.09) 234	−0.10 (0.09) 234
Attention/Impulsivity (AR)	0.08+ (0.04) 329	0.07+ (0.04) 329	0.13^*^ (0.06) 233	0.11^*^ (0.05) 233
Teacher survey of classroom behavior				
Regulation-related skills (CEFS)	0.14+ (0.08) 465	0.13+ (0.08) 462	0.08 (0.08) 413	0.07 (0.07) 410
Prosocial behavior (TOCA-R)	0.04 (0.09) 465	0.04 (0.09) 462	0.25^*^ (0.08) 413	0.24^*^ (0.08) 410
Teacher-student relationship (TSRS)	0.11 (0.10) 453	0.09 (0.09) 450	0.13 (0.13) 413	0.08 (0.11) 410
Discipline events	0.03^*^ (0.01) 483	0.03^*^ (0.01) 480	0.02 (0.02) 431	0.02 (0.02) 428
Controls				
Child-level	No	Yes	No	Yes
Classroom-level	No	No	Yes	Yes

Estimate (S.E.) with *N* underneath for each outcome. *N* is reduced with the inclusion of classroom covariates; PT, Pencil Tap; DCCS, Dimensional Change Card Sort; TMT, Trail Making Task; FDS, Forward Digit Span; BDS, Backward Digit Span; PSRA-AR, Preschool Self-Regulation Assessment – Assessor Report; CEFS, Child Executive Function Survey; TOCA, Teacher Observation of Classroom Adaptation; and TSRS, Teacher Student Relationship Scale.

#### Teacher Survey of Classroom Behavior

As shown in the bottom panel of Table 5, there were no statistically significant impacts of the BG on children’s prosocial behavior or the overall quality of teacher–student relationships in Models 1 and 2 (without and with child demographic covariates, respectively). There was a marginally significant impact of the BG on teacher-reported regulation-related skills measured by the CEFS (*b*=0.13, *S.E.* =0.08, *p* <0.10) and a negative effect of the BGs on teacher-reported discipline (*b*=0.03, *S.E.* =0.01, *p*<0.05), but we note that there were relatively few instances of disciplinary infractions making this estimate likely quite imprecise. As shown in the bottom panel of Table 5, Model 4, while there were no statistically significant impacts of BG on the overall quality of teacher–student relationships, children’s regulation-related skills, or discipline events, we did observe impacts for children’s prosocial behavior as reported by their teachers, *b*=0.16, *S.E.* =0.07, *p*<0.05, ES=0.24.^3^

## Discussion

Despite the immense interest and investment in EFs and related self-regulation skills in school contexts, findings thus far are equivocal, resulting in the need for additional compelling evidence from rigorous studies of school-based interventions. This paper contributes to the relatively sparse body of literature on non-curricular, strategy-based approaches designed to cultivate EFs and related self-regulation skills in classroom contexts, providing additional evidence for the feasibility and potential efficacy of these interventions (e.g., McClelland et al., 2019). The goal of this study was to evaluate the impact of the BG, a game-based, classroom-level intervention designed to build preschool and elementary-aged children’s EFs and related SR skills, on children’s outcomes. The study was designed to address a number of limitations described above by including a relatively large sample, a diverse measurement approach, classroom-level randomization, and appropriate analytic techniques that account for the nesting of students in classrooms.

Evidence presented in this article from Model 2 (with child covariates) indicate marginal improvements in teacher-reported regulation-related skills and observer-reported attention and impulsivity, though all marginal effects should be interpreted with caution. Model 2 also shows decreases in global EF as measured by the DCCS direct assessment (though we note that this finding is not robust to an alternative scoring approach) and increases in disciplinary incidents. Model 4^4^ (including child and classroom covariates that are linked to teacher implementation and child outcomes) indicates improvements in children’s assessor-reported attention and impulsivity, global EF as measured by a direct assessment (the TMT), and teacher-reported prosocial behavior. The magnitude of effects in Model 4 is notable, an effect size of nearly 1/2 a SD (ES=0.41) for the TMT, nearly 1/3 of a SD for the AR (ES=0.27), and 1/4 of a SD for the TOCA-R. It is notable that we detect effects on multiple types of measures (teacher report, observer report, and direct assessment), with an intervention delivered by teachers after a short 90-minute training minimally trained teachers, who weave the work into their own practice (e.g., throughout the day to the degree they can), and that the effects are similar to (and even larger than) some comprehensive interventions (Durlak et al., 2011). Together, the estimates from Models 2 and 4 provide preliminary evidence for the impact of BG on children’s outcomes, suggesting there is a signal that implementation of BGs in classroom contexts can potentially improve children’s outcomes. However, this signal should be investigated further with larger and more tightly controlled designs.

An interesting pattern of findings emerged for the teacher report of regulation-related skills and discipline events. In the first two models, one without any covariates and one with child covariates, teachers rated children in the intervention group as having marginally higher regulation-related skills, but more disciplinary events than their peers in the control group. While initially counterintuitive, there are a few possible explanations. After attending the BG training and learning more about the links between EF, self-regulation, and behavior, teachers in the intervention group may be more attuned to the regulatory behaviors included in the regulation-related skills survey (e.g., remembering lists or items in the correct order). At the same time, they may also be more likely to notice when students *are not* demonstrating these skills, perhaps leading to increased disciplinary action. However, with the inclusion of classroom covariates, there were no remaining statistically significant differences between the intervention and control groups on these measures, suggesting that disciplinary incidents may be more closely linked to the composition of children in the classroom rather than a byproduct or impact of the intervention. The lack of effects on the regulation-related skills survey could be explained by the highly controlled nature of the classroom environment in these charter schools, which provide few opportunities for students to practice regulating their own behavior (e.g., see Bailey et al., 2019a).

Similarly, in the first two models (no covariates and child covariates only) there was a significant, negative impact of the intervention on children’s global EF as measured by the DCCS. As above, with the inclusion of classroom covariates, these findings were no longer statistically significant. This is puzzling as there was a significant and positive impact of BG on the other measure of global EF, the TMT, in the final two models (classroom covariates only and combined child and classroom covariates). Though both measures assess global EF, they differ in what they ask children to do. The DCCS is a sorting activity with a high-level of involvement from the assessor who presents each card for the child to sort. On the other hand, the TMT involves more independence for the child as they work on their own to link numbers, letters, and a combination of the two, with the assessor present in a supervisory capacity. The TMT may more closely mirror the independent work of the classroom and the ways in which children practice deploying their EFs in context (particularly in this academically focused charter network).

Though we leave it to the reader to make their judgement, we find Model 4 compelling for two key reasons. First, the decisions teachers make about intervention implementation (e.g., amount of game play) influence children’s outcomes (e.g., Humphrey et al., 2018) and are influenced by their perception of the students in their classrooms. As described above, teachers were more or less likely to implement BG based on certain classroom characteristics. For example, classrooms with more students or more students coming from homes in which English is not the primary language tended to play more games and classrooms with more students perceived as performing below grade level tended to play fewer games. Many of these classroom characteristics are also statistically significantly correlated with children’s outcomes (see Table 4). As noted above, the absence of a scope and sequence provides teachers with the autonomy to decide if, when, and how they implement the BG. Including these classroom covariates in our analysis accounts for the role that teacher perception of the classroom environment and the characteristics of the students within it play in intervention implementation and students’ outcomes.

An important consideration for interpreting these findings is the association between bilingualism and executive function, particularly in Latinx populations. Foundational and interconnected executive function and language skills develop bidirectionally (i.e., growth in one skillset promotes growth in the other) beginning very early in life (Bialystok, 2015; Hanno and Surrain, 2019). Studies have shown that Spanish–English bilingual students tend to have higher levels of EF skills and perform better on EF tasks from early childhood through adulthood (Riggs et al., 2014; White and Greenfield, 2017). As such, one possible explanation for some of the null findings in this primarily Latinx sample (95%) is that EF tends to be an area of strength for Latinx students, and that use of BG reinforced, but did not significantly grow, students’ already high EF skills. In addition to testing BG in diverse samples, future research should include data on bilingualism to better understand the interaction between bilingualism and BG impacts.

While this study has several strengths, a number of limitations should be noted. First, although classroom-level randomization allowed us to account for between-school differences, there is potential for spillover effects between classrooms. This is especially possible in the older grades, where the floater teacher spent time in both the intervention and control classrooms. Though we have no evidence that spillover effects occurred, it is possible that impacts were attenuated if teachers in the control group received any materials or information from their peers. Second, although the BG promote autonomy and are easy to integrate into daily activities and routines, the charter network followed a strict schedule, with very little room for integration. In contrast with previous studies of BG where teachers could easily implement the games into any part of their daily routine (e.g., Jones and Imm, 2016), it may have been more challenging for teachers in the charter network to find time to play the games on a daily basis. Third, although we began to explore BG use in a descriptive sense, our study did not include a measure of implementation fidelity. It is possible (and likely) that in addition to amount of game play, quality of game play and adherence to key aspects of the BG structure (e.g., reflection questions) may contribute to the effectiveness of the intervention. Future research should include measures of implementation fidelity. Furthermore, we plan to follow this paper with one addressing key mechanisms, including the role of implementation/exposure on children’s outcomes, and the degree to which any treatment induced changes in executive function and self-regulation underlie any observed changes in children’s behavior. Fourth, missing teacher survey data reduced the analytic sample in the final two models, which included classroom covariates. Though there were no significant differences between students with and without classroom covariates on child demographic characteristics (gender, SPED, or ELL status), it is possible that there were differences between classrooms in which the teacher survey was completed vs. those in which it was not. Fifth, though multiple EF measures are a strength of the study and no more than four outcomes were tested within the same sample (e.g., direct assessments were only completed with a subsample of children and measures differed between grade bands), multiple comparisons, or the detection of a significant effect by chance, are possible and a potential limitation. Finally, we note that this study was underpowered, which can influence how we understand the null findings. With more power, we may have been able to detect additional effects of the intervention on children’s outcomes.

In summary, our findings contribute to the existing literature in three key ways. First, our results provide support for the feasibility of low-cost, easy to implement intervention approaches to foster social and emotional skills in school settings (see Jones et al., 2018; Bailey et al., 2019b). The minimal training and support provided to teachers combined with the freedom to decide the details of game play (e.g., which game to play, when, how to adapt) mirrors a programmatic approach in the typical school environment (outside of an intervention study), underscoring the feasibility of the intervention and the possibility for sustainability and scale. Second, when considering the broad correlational effects of EFs and positive short- and long-term outcomes (e.g., Jacob and Parkinson, 2015), particularly for high-risk children like those in this sample, the BG and similar interventions could be a route to improving overall academic engagement and behavior, though given the mixed findings, this should be investigated further. An important next step in our work on BG and similar interventions is to explicitly test the impact of these interventions on academic skills. Third, our findings suggest the potential benefits of a targeted game-based intervention designed to build EFs and related self-regulation skills in preschool and elementary classroom contexts. Findings from our models with child covariates suggest decreases in global EF as measured by the DCCS (not robust to scoring approach), increases in disciplinary incidents, and marginally significant improvements in attention and impulsivity. Findings from our models with child and classroom covariates indicate improvements in children’s global EF (measured by direct assessment, TMT), prosocial behavior (measured by teacher survey), and attention and impulsivity (measured by assessor report) for students who received the BG intervention. Findings from our models with child and classroom covariates suggest decreases in global EF as measured by the DCCS (not robust to scoring approach) and increases in disciplinary incidents. Though this study addresses the need for more robust, multi-informant measurement approaches in previous studies (e.g., Crooks et al., 2020), additional studies with larger samples and more tightly controlled designs are needed to confirm the signal we observe on the effects of BG in classroom contexts. Finally, the development of BG across trials and the small-scale RCT presented in this paper provides a model for how interventions and what we know about their effectiveness should be built in phases across multiple trials that increase in size, scope, and rigor. The next step of evidence building for the BGs will include a larger randomized trial, ideally in a more diverse and generalizable set of schools.

## Data Availability Statement

The datasets presented in this article are not readily available because data may be shared in certain situations, with PI approval. Requests to access the datasets should be directed to stephanie_m_jones@gse.harvard.edu.

## Ethics Statement

The studies involving human participants were reviewed and approved by Harvard’s Committee on the Use of Human Subjects (CUHS). Written informed consent to participate in this study was provided by the participants’ legal guardian/next of kin.

## Author Contributions

SB, RB, and SJ contributed substantively to key elements of this work from original conceptualization. RB and SJ contributed to the design of the BGs. SJ, SB, and RB contributed to funding. SB and SJ contributed to study design and execution, data management, analysis, and write-up. All authors contributed to the article and approved the submitted version.

## Funding

This project was funded by the Dean’s Venture Fund, Harvard Graduate School of Education of Education, Harvard University.

## Conflict of Interest

The authors declare that the research was conducted in the absence of any commercial or financial relationships that could be construed as a potential conflict of interest.

## Publisher’s Note

All claims expressed in this article are solely those of the authors and do not necessarily represent those of their affiliated organizations, or those of the publisher, the editors and the reviewers. Any product that may be evaluated in this article, or claim that may be made by its manufacturer, is not guaranteed or endorsed by the publisher.

## Footnotes

^1^As a scoring robustness check, we also tested a continuous scoring approach (e.g., McClelland et al., 2014), where the number of correct cards across all rounds were summed to create a final score.

^2^While there is literature to suggest this categorization (i.e., Hongwanishkul et al., 2005; Zelazo, 2006), some might disagree (e.g., Karalunas et al., 2016 employed the DCCS as a measure of working memory), illustrating the complexity of operationalizing EFs.

^3^Length between fall and spring assessments ranged from 211 to 268 days. This “length” variable was included in Model 4 as a robustness check. Results were robust to the inclusion of the “length” variable, with the exception of Trail Making Task that became marginally significant.

^4^We note again the samples differ between Models 2 and 4 [due to greater missing data in classroom level covariates, but that these two samples do not differ from each other significantly in any child-level factors (demographic characteristics and baseline outcomes)]. As a result, standard errors are slightly larger in Model 4.

## Appendix

Appendix baseline equivalence for reduced sample with non-missing classroom covariates.

**Table:**

	Full sample			Intervention group				Control group				Stand. mean dif.
	*N*	Un adjusted mean	Pooled SD	*N*	Mean	Adj. mean	SD	*N*	Mean	Adj. mean	SD	
Direct assessments of children’s EF and related self-regulation skills												
Pre-K and kindergarten												
Inhibitory control (Pencil Tap)	47	0.82	0.23	28	0.78	0.75	0.27	19	0.87	0.83	0.15	0.32
Grades 1–4												
Global EF (Trail Making Task)	172	2.74	0.59	90	2.70	2.28	0.63	82	2.79	2.32	0.54	0.05
Short term memory (Forward Digit Span)	174	4.51	1.01	90	4.50	3.94	0.96	84	4.53	4.06	1.08	0.12
Working memory (Backward Digit Span)	174	3.93	1.10	90	3.93	3.21	0.91	84	3.93	3.35	1.28	0.12
All students												
Global EF (Dimensional Change Card Sort)	234	2.22	0.71	127	2.21	1.87	0.72	107	2.22	1.83	0.69	0.06
Attention/Impulsivity (Assessor Report)	233	2.60	0.44	126	2.55	2.33	0.47	107	2.66	2.43	0.40	0.23
Teacher survey of classroom behavior												
Regulation-related skills (CEFS)	410	2.81	0.85	250	2.80	2.64	0.82	160	2.83	2.70	0.91	0.06
Prosocial behavior (TOCA)	410	4.31	0.68	250	4.34	4.45	0.70	160	4.26	4.37	0.65	0.13
Teacher–student relationship (TSRS)	410	3.35	0.78	250	3.37	3.25	0.80	160	3.30	3.22	0.74	0.04
Discipline events	428	0.07	0.23	250	0.09	0.11	0.24	178	0.05	0.08	0.20	0.17
Demographic characteristics												
Gender (female=1)	428	0.50	0.50	250	0.51	0.53	0.50	178	0.48	0.50	0.50	0.08
SPED	428	0.11	0.31	250	0.10	0.08	0.30	178	0.12	0.11	0.32	0.09
ELL status	428	0.41	0.49	250	0.42	0.37	0.49	178	0.40	0.41	0.49	0.17

*F*-test for overall balance on the pre-K and K measure of inhibitory control (Pencil Tap) revealed no statistically significant difference between students assigned to intervention vs. control groups (*F* (3, 7)=1.49, *p*=0.2974). *F*-test for overall balance on direct assessments in Grades 1–4 revealed no statistically significant difference between groups assigned to intervention vs. control groups (*F* (5, 14)=0.28, *p*=0.9162). *F*-test for overall balance on direct assessments used in all grades revealed no statistically significant difference between students assigned to intervention vs. control groups (*F* (7, 26)=0.79, *p*=0.6011). *F*-test for overall balance on initial balance in the teacher survey of classroom behavior revealed no statistically significant difference between groups assigned to intervention vs. control groups (*F* (9, 25)=0.83, *p*=0.5933). *F*-tests included school fixed effects and clustered standard errors at the classroom level.
